# Evaluating the impact of virtual reality game training on upper limb motor performance in children and adolescents with developmental coordination disorder: a scoping review using the ICF framework

**DOI:** 10.1186/s12984-024-01393-y

**Published:** 2024-06-05

**Authors:** Mohammed Alharbi, Haoyang Du, David Harris, Greg Wood, Helen Dodd, Gavin Buckingham

**Affiliations:** 1 Department of Public Health and Sport and Sciences, University of Exeter, Exeter, UK; 2 Department of Physical Therapy, Faculty of Applied Medical Sciences, University of Hail, Hail, Kingdom of Saudi Arabia; 3 Department of Sport and Exercise Sciences, Manchester Metropolitan University, Manchester, UK

**Keywords:** DCD, Dyspraxia, Paediatric, VR

## Abstract

**Objective:**

This scoping review aims to explore published literature testing Virtual Reality (VR) interventions for improving upper limb motor performance in children and adolescents with Developmental Coordination Disorder (DCD). Our primary focus was on the types of VR systems used and the measurement tools employed within the International Classification of Functioning, Disability and Health Children and Youth Version (ICF-CY) domains in these studies.

**Methods:**

A comprehensive search of six electronic databases up to 11th January 2024 was conducted using predefined terms. Inclusion and exclusion criteria were applied to determine study eligibility, with two authors independently assessing titles, abstracts, and full-text articles.

**Results:**

Out of 788 potential studies, 14 met the eligibility criteria. Studies predominantly utilized non-immersive VR (nVR) systems, for example, commercial platforms such as Nintendo Wii. Most interventions targeted general motor coordination or balance, with only four studies specifically focusing on upper limb motor performance. The Movement Assessment Battery for Children-2 was the predominant assessment tool. However, the use of game scores and trial durations raised concerns about the accuracy of assessments. The majority of studies reported no significant improvement in upper limb motor performance following VR interventions, though some noted improvements in specific tasks or overall outcomes.

**Conclusion:**

The findings suggest that, while nVR interventions are being explored for paediatric motor rehabilitation, their impact on enhancing upper limb motor performance in children with DCD is unclear. The variability in intervention designs, outcome measures, and the predominant focus on general motor skills rather than specific upper limb improvements highlight the need for more targeted research in this area.

**Impact:**

This review underscores the importance of developing precise and clinically relevant measurement tools in a broader range of VR technologies to optimize the use of VR in therapy for children with DCD. Future research should aim for more rigorous study designs and emerging immersive technologies to maximize therapeutic benefits.

**Supplementary Information:**

The online version contains supplementary material available at 10.1186/s12984-024-01393-y.

## Introduction

Developmental coordination disorder (DCD), also known as developmental dyspraxia, is a chronic and usually permanent condition prevalent in 5–6% of children [1]. These children experience numerous functional difficulties due to motor coordination [2]. A primary concern in many children and adolescents with DCD is impaired upper limb function, which particularly affects their ability to perform Activities of Daily Living (ADLs) using their hands, fingers, and arms [3]. Common ADLs that pose challenges include essential self-care tasks such as dressing, tying shoes, eating with utensils, and personal hygiene activities such as brushing teeth and hair. These difficulties can significantly impact children’s psychological wellbeing [4, 5]. Consequently, there is often a limitation in social participation [6], a decrease in the independence of affected individuals, and a reduction in their overall quality of life [7].

The World Health Organization (WHO) framework, the International Classification of Functioning, Disability and Health Children and Youth Version (ICF-CY), provides a comprehensive structure for understanding health and disability in children and adolescents [8]. This framework classifies health components into body functions and structures, activities and participation, and contextual factors, which include personal and environmental factors [8]. Specifically, the ICF-CY emphasizes the importance of 'Body Functions' as physiological processes of body systems, which for children and adolescents with DCD, pertains critically to upper limb function. While 'Body Structures' such as arms and hands are typically anatomically with no issue in children with DCD, these structures are involved in daily activities that can be challenging due to impaired motor skills [8]. Therefore, although the anatomical structures are not affected, the functionality and effective use of these structures in performing tasks are compromised. 'Activities' involve the execution of tasks, such as using utensils or dressing, where impaired upper limb function can present significant barriers [8]. These activities highlight the practical challenges faced by children with DCD, as their condition does not affect the structure of their limbs, but rather their ability to coordinate and control movements effectively [8]. 'Participation,' defined as involvement in life situations, can be severely limited by difficulties in these activities, impacting educational opportunities and social interactions [8].

Motor challenges in upper limb function restrict not only basic ADLs but also the ability to engage in leisure and recreational activities, which are a key aspect of social integration [7]. Addressing these challenges through targeted interventions can minimize long-term impacts and improve motor performance across various life domains [9]. Consequently, without timely and appropriate therapeutic intervention, these difficulties may persist into adulthood, affecting broader community life and functioning [10]. Early intervention that focuses on enhancing upper motor function is therefore essential to promote independence in daily living, self-care, and play, especially in educational settings for children and adolescents with DCD.

Conventional rehabilitation strategies for children and adolescents with DCD, based on basic motor learning concepts, often emphasise repetitive physical practice of tasks or task components [11]. However, this approach requires long trials and/or extensive repetition of training [12] which may lead to boredom and reduced motivation [13]. Recently, there has been growing interest in exploring more engaging and enjoyable alternatives, such as Virtual Reality (VR) [14].

VR, a technology that encourages full-body movement to interact with an immersive computer-generated environment, is rapidly becoming significant part of consumer entertainment [15]. In the context of rehabilitation, VR offers a motivational, naturalistic environment with immediate feedback [14, 16], potentially leading to higher treatment adherence [17] and increased movement repetition [18, 19]. Factors such as enjoyment and motivation are critical in influencing children's participation and success in intervention programs [20]. By enhancing these factors, VR can transform learning into a rewarding process, inspiring children and adolescents to actively participate, explore, and persist in the tasks they are given [21]. Thus, VR-based play activities for paediatric rehabilitation may be especially beneficial, providing playful exploration opportunities while mitigating impairment effects and enhancing compliance with repetitive practice necessary for skill acquisition [22].

Reviews have increasingly explored the use of VR-based interventions across various paediatric populations [22–30], and a number of reviews have been performed to determine the effect of such interventions [24, 29, 30]. These reviews generally indicate that VR interventions appear promising in children and adolescents, suggesting potential benefits in motor skill enhancement and rehabilitation. To date, three review articles have evaluated the impact of VR interventions on children with DCD, examining general motor performance, with a strong focus on balance. For example, in the review by Cavalcante et al. [31], out of 12 studies examining VR's impact on motor performance, nine exclusively utilized balance games through the Wii-balance board, highlighting a gap in addressing upper limb functions crucial for daily activities. The two other reviews in this area have examined the impact of VR for improving motor skills in children with neuromotor dysfunction, including DCD [32, 33]. Although they examined multiple cohorts including CP and Down syndrome, one of these studies, however [32], specifically excluded outcomes related to fine motor skills, such as the Movement Assessment Battery for Children-2 (MABC-2) manual dexterity component, thereby limiting its scope to gross motor functions. Similarly, Mentiplay et al. [34] also excluded studies focusing solely on upper limb function outcomes. As a result, the conclusions drawn in these reviews about the effects of VR on upper limb function, may not provide a comprehensive understanding of how VR can facilitate the training and improvement of upper limb movements.

Since its initial introduction in the 1990s, the application of VR in rehabilitation has seen considerable growth and development [22]. While immersive VR (iVR) headsets (e.g., the Meta Quest 2) are a reasonably new consumer product, non-immersive VR (nVR) has existed for almost two decades and researchers have examined the efficacy of these products in a number of contexts [30, 35]. Given the diversity of VR hardware and software available in recent years, assessing the overall evidence base for this range of technologies is challenging. This review classifies studies using a technology taxonomy to distinguish between iVR and nVR systems. This distinction is crucial, as each system offers distinct modes of perception–action coupling, essential for developing dextrous skills, a core issue for children and adolescents with DCD [36, 37]. iVR systems, characterized by head-mounted displays and motion-tracking controllers, provide a unique immersion level and potentially superior perception–action coupling compared to nVR systems, which involve traditional screen-based interaction with controllers (e.g., Nintendo Wii) [38]. The impact of these VR systems may vary depending on whether the hardware and software were designed for rehabilitation or entertainment [39]. Literature that fails to differentiate between these VR types may risk distorting our understanding the potential value of VR in training and rehabilitation.

Despite the increasing interest in VR as an innovative intervention for paediatric rehabilitation [40], especially for children and adolescents with DCD, the literature shows a notable focus on motor skills involving balance, with limited attention to upper limb functions. Moreover, previous reviews in this area have typically included broad populations with various neurological conditions, often excluding studies that specifically address upper limb motor skills in children with DCD. Thus, there is a significant gap in understanding how VR can be used to improve these crucial aspects of motor performance.

To address this gap, our scoping review aims to provide a comprehensive exploration of VR interventions targeted at enhancing upper limb motor performance in children and adolescents with DCD. The scoping review methodology was chosen for its flexibility in mapping the range of available evidence and identifying gaps in the literature. Unlike systematic reviews, which typically focus on specific outcomes and apply rigorous inclusion criteria, scoping reviews allow for a broader exploration of existing studies, enabling us to examine a wide range of VR systems, tools, and intervention strategies, which is particularly important given the rapid pace of technological development in this field. By exploring the scope of the existing research, this review will provide a clearer understanding of the current landscape and offer insights into future research directions, ultimately contributing to improved therapeutic interventions for children with DCD.

## Materials and methods

The following stages were followed to conduct this scoping review [41, 42]: (1) Identifying the specific query or problem that the research aims to investigate; (2) identifying pertinent research papers; (3) selecting the appropriate studies; (4) organizing the data; and (5) compiling, summarizing, and presenting the findings. Taking into account the PRISMA-ScR reporting guidelines [43], this review was performed according to the JBI methodology for scoping reviews [44]. The protocol of this scoping review was registered on the Open Science Framework: https://osf.io/s938w/?view_only=935793cd619646f9a14187a1bcade6eb

There were minor deviations from the initial protocol, specifically regarding the refinement of the research questions to better align with the objectives of a scoping review. Additionally, we expanded the focus of the review to comprehensively address all domains of ICF-CY. This adjustment was made to ensure a more thorough exploration of how VR interventions impact various aspects of functioning and participation in children with DCD.

### Identification of the research question

The primary aim of this scoping review is to comprehensively explore the use of VR as an intervention for improving upper limb motor performance in children and adolescents with DCD. To achieve a thorough understanding of this field, our review is guided by the following research questions:

1. What is the reported range and nature of VR interventions for improving upper limb motor performance in children and adolescents with DCD? This question seeks to delineate the various VR interventions that have been employed, focusing on their characteristics, methodologies, and targeted outcomes. As part of this exploration, a key sub-question is: What types of measurement tools are being used in these VR interventions to assess upper limb motor performance in children and adolescents with DCD within the domains of the ICF-CY? This sub-question aims to identify and evaluate the tools and methods used to measure the efficacy of VR interventions, which is crucial for understanding their impact and applicability.
2. What specific types of VR systems, including iVR and nVR, have been utilized in studies focusing on upper limb motor performance in children and adolescents with DCD? This question explores the technological aspect of VR interventions, aiming to identify and describe the range of VR systems employed across these studies.
3. How do VR interventions influence the motivation and enjoyment of children with DCD during rehabilitation sessions?

### Identification of relevant studies

A search was carried out across the following electronic databases, from their establishment up until 21st of January 2023 and updated on the 11th of January 2024: EMBASE (via Ovid), Medline (via Ovid), PubMed, Web of Science, The Cochrane Central Register of Controlled Trials (CENTRAL), CINAHL (via EBSCOhost), and Google Scholar. The approach used to find relevant studies was using appropriate keywords and specific medical terminology known as Medical Subject Headings (MeSH) to enhance the relevance of the search which was developed by the research team. A full illustration of the search method used in the EMBASE database, which was later customized to meet the requirements of other databases, is shown in Appendix 1. The included studies and related systematic reviews were checked by hand-searching their reference lists for any other relevant literature that might have been missed. In situations where it was clear that the studies met the inclusion criteria, or when additional clarification was required to establish their eligibility, a full text examination was undertaken. There were no restrictions placed on the publication date.

### Study selection

Studies were considered eligible for inclusion if they satisfied the following criteria: (1) had collected data from a participant population who consisted of individuals aged 18 years or younger of either gender; (2) Participants were either previously diagnosed with DCD or met the criteria recognised by the Diagnostic and Statistical Manual of Mental Disorders, Fourth and Fifth Editions (DSM-IV and DSM-V), for the condition, or they scored at or below the 16th percentile on the MABC-2; (3) measured upper-limb motor performance; (4) employed games focussed on upper limb movement; (5) used iVR or nVR; and (6) were published in English language. All types of study design were included.

Studies were excluded if: (1) they recruited adult participants aged > 18 years old; (2) included children and adolescents with other neurological conditions affecting motor performance such as Cerebral Palsy (CP); (3) they were published in a language other than English; (4) were non-original, non-full-text research such as abstracts, and commentaries; (5) VR was not the main intervention program; (6) a study did not measure the impact of the intervention on upper extremity performance; or (7) were a non-empirical report: meta-analysis; review; commentary.

Covidence, a tool for producing systematic reviews [45], was initially employed by the research team to facilitate the analysis of the articles. For the updated search conducted in 2024, we utilized Rayyan, a web-based application specifically designed for systematic reviews [46]. A notable difference between these two tools is that while Covidence offers automatic duplication removal, Rayyan requires researchers to manually identify and remove any duplicated studies. The outcomes from the database searches were exported to both Covidence and Rayyan for processing. After removing duplicates, two independent reviewers (MA and HD) analysed the article titles and abstracts using our pre-defined inclusion criteria to determine their eligibility. This initial screening was followed by a more detailed examination of the full texts of the studies that met the initial criteria. During this stage, the inclusion and exclusion criteria were again applied to further refine the selection of studies. Once this comprehensive screening and full-text review process was completed, the remaining studies were deemed appropriate for inclusion in our review.

### Charting the data

Two independent reviewers utilized a predetermined content field excel spreadsheet for data extraction from the included studies. This process was carried out in parallel, with each reviewer working independently to minimize potential bias.

Subsequently, the main author cross-checked the extracted data against the full text of the included studies, verifying the accuracy of the information gathered. The data extraction process adhered to the guidelines for conducting systematic scoping reviews as established by Peters et al. [44]. The extracted data encompassed the following key elements:

- First author, publication year, and study design of each study
- Study population, sample size, and comparison groups
- MABC-2 percentile
- VR tool utilized in the study
- Intervention protocols and comparators
- Outcome measures used to assess efficacy and ICF-CY domains
- Results pertaining to upper limb motor performance

In instances where discrepancies were identified in the extracted data, the full-text article was revisited, and a consensus was reached through discussion among the reviewers. This process ensured that the data extraction was comprehensive and reliable, providing a solid foundation for the subsequent analysis and synthesis of the scoping review findings.

A critical appraisal of the included studies was not conducted in this review. According to the Arksey and O'Malley [41] framework for carrying out scoping reviews, such an appraisal is not deemed necessary. Furthermore, this approach has been acknowledged and endorsed by the database of scoping reviews pertaining to health-related subjects [43].

### Collating, summarizing, and reporting findings

In our scoping review, we focused on identifying and organizing key themes derived from the data, concisely presented in Table 1. The themes were primarily centred around four aspects: the type of VR tools used, the specific protocols of the interventions, the variety of outcome measures employed, and the overall impact of VR on upper limb motor performance in children and adolescents with DCD. We categorized VR tools into immersive and non-immersive systems to understand the range of technologies applied. Intervention protocols were analysed in terms of session duration, frequency, and intensity, offering insights into the operational aspects of these VR interventions. For outcome measures, our focus was on both motor competence and functional performance tools, assessing how effectively these measures capture improvements in motor skills. Lastly, we summarized the impact of the VR interventions, noting trends in successful outcomes, mixed results, or lack of efficacy as reported in the studies. This structured approach allowed us to provide a clear overview of the current research landscape in VR interventions for DCD, highlighting promising areas and gaps that warrant further investigation.

**Table 1:** The key features of the included studies

Study/design	Study population, sample size and comparison groups	MABC-2 percentile	VR tool	Intervention protocols and comparator	Outcome measures and ICF-CY domains	Results regarding upper limb motor performance
Ashkenazi et al. [50] RCT	30 children with DCD (25 boys), aged 4 to 6 years They were randomly assigned to either: A. VR group (n = 15) B. Usual care group (n = 15)	≤ 15	Sony PlayStation 2 EyeToy	Group A: children play multiplayer games with a parent Group B: usual physiotherapy treatment	• MABC-2 ICF-CY domains: activities • DCD-Q ICF-CY domains: activities	• No significant changes in both MABC-2 and DCD-Q were observed
Ashkenazi et al. [51] Pilot feasibility study	9 children with DCD (7 boys), aged 4 to 6 years	≤ 15	Sony PlayStation 2 EyeToy	The intervention consisted of 2 parts: A. VR-based intervention (45 min): the children played up to 4 single-player games during each of the first 4 weeks of VR sessions. During sessions 5 to 10, the children also played multiplayer games with a parent B. Goal-directed task (15 min): task-specific non-VR activities were carried out in accordance with each child’s stated goals such as riding their own bicycles, playing ball games (soccer, basketball), or putting on and taking off their clothes	• MABC-2 ICF-CY domains: activities • DCD-Q ICF-CY domains: activities	• No significant changes in both MABC-2 and DCD-Q were observed
Bonney et al. [52] RCT	43 girls with DCD aged 13–16 years They were randomly assigned to either: A. TFT group (n = 22) B. Wii training group (n = 21)	≤ 16	Nintendo Wii	Group A: each session consisted of 10 min of group dance, 25 min of motor skills instruction and in the final 10 min, the participants engaged in popular games such as capture the flag, traffic cop and netball Group B: each participant played a maximum of eight games per session	• MABC-2 ICF-CY domains: activities	No significant deference found
Cavalcante neto et al. [47] RCT	76 children, of which 36 children with DCD, aged 7–10 years Total boys (DCD + TD) = 42 They were randomly assigned to either: A. Wii group (n = 16 DCD; 20 TD) B. Kinect group (n = 19 DCD; 21 TD)	≤ 16	Nintendo Wii and Microsoft Kinect	Group A: children played 10 different games twice, spread over two days per week Group B: children played 9 different games, with the first five games on the first training day and the last four on the second day of the week. Children played each one of the first five games twice, while each one of the last four games were played only once due to the length of the game	• Games performance scores ICF-CY domains: activities	No differences were found in game score between the TD and DCD group
Cavalcante neto et al. [54] RCT	32 children having DCD (24 boys), aged 7–10 years They were randomly assigned to either: A. Wii group (n = 16) B. TST group (n = 16)	≤ 16	Nintendo Wii	Group A: played 6 different games Group B: played 6 games that were closely matched to A group	• MABC-2 ICF-CY domains: activities	Pre–post training effects for each group: there was no significant pre–post change on manual dexterity or aiming/ catching components for either group Comparison of (pre–post) change scores between groups: no significant group difference
Cavalcante neto et al. [59] RCT	32 children having DCD (24 boy), aged 7–10 years They were randomly assigned to either: A. Wii group (n = 16) B. TST group (n = 16)	≤ 5	Nintendo Wii	Group A: played 4 games, table tennis frisbee, archery and bowling, related to upper limb Group B: 4 games were closely matched to the Wii group	• Games performance scores ICF-CY domains: activities	The Wii training group showed greater improvement than the TST group in some games (archery and bowling)
Ferguson et al. [55] Quasi experimental Study	46 children with DCD (24 boys), aged 6–10 years They were assigned to either: A. NTT group (n = 27; 15 boys) B. Wii group (n = 19; 9 boys)	≤ 16	Nintendo Wii	Group A: this training sessions was conducted as a group between 5 to 8 children. Games included activities such as soccer, netball, variations of tagging games and other indigenous games popular amongst children from this community Group B: children played 13 various games, 5 of them incorporating arm movements. Children were instructed to choose one of these games and play it twice before choosing a different game	• MABC-2 ICF-CY domains: activities • FSM ICF-CY domains: not categorized • HHD ICF-CY domains: not categorized	MABC-2: significant differences for the NTT group compared to the Wii group were seen in manual dexterity. On examination of the aiming and catching component scores, no significant effects for both groups were noted FSM & HHD: no significant effects for both groups were noted
Gonsalves et al. [62] Quasi experimental Study	38 children aged 10–12 years They were assigned to either: A. DCD group (n = 21; 11 boys) B. TD group (n = 17; 9 boys)	≤ 16	Sony PlayStation 3 Move and Microsoft Kinect	Both groups played table tennis on Move for 5 practice points, followed by one full game to 11 points. This process was then repeated using Kinect	• Kinematic performance to measure: *1. Hand path measures* (distance, duration, average speed and maximum speed for each forehand and backhand) ICF-CY domains: not categorized 2. *Wrist and Elbow Angle Variables* (maximum and minimum angle for each forehand and backhand) ICF-CY domains: not categorized	• *hand path measures:* Between groups (DCD and TD) comparison: children with DCD utilized a significantly slower maximum hand speed than children with TD during the backhand strokes regardless of video game type although there were no significant differences in hand path distance • *Wrist and Elbow Angle Variables:* Between groups (DCD and TD) comparison: significant differences were found for maximum and minimum wrist angles during both forehand and backhand strokes. These results of the maximum and the minimum wrist angles showed that children with DCD played table tennis on both consoles with significantly more wrist extension than children with TD
Smits-engelsman et al. [56] RCT	50 children, of which 16 (7 boys) children with DCD, aged 6–10 years They were randomly assigned to either: A. Ball Wii B. Agility Wii (8 DCD and 17 TD per group)	≤ 16	Nintendo Wii	Group A: played virtual Ball games that seem related to real-world ball games such as golf, baseball and tennis Group B: played virtual agility games such as Snowboard, Obstacle course and Skateboard Reflected on Real-World Games: it included practice of fundamental movement skills, which are required to perform aiming and catching activities	• MABC-2 ICF-CY domains: activities • BOT-2 ICF-CY domains: activities • PERF-FIT ICF-CY domains: activities	Ball Skills & Agility Skills: no significant effects between the TD and DCD children for both groups were noted *Real-world Game scores:* the children improved on the scores of the real-world games but transfer of learning from a virtual environment to real-world contexts does not appear to be very task specific
Snapp-childs et al. [61] Quasi experimental study	16 children aged 7–8 years They were assigned to either: A. DCD group: 8 children (3 boys) with DCD B. TD group: 8 TD children	N/A	Phantom Omni	The training started with the highest level of magnetic attraction, slowest competitor, and shortest path. Once the participant beat both competitors, he or she then moved to the next longest path (with slowest competitor). After all paths and competitors were ‘‘beaten,’’ the level of magnetic attraction was decreased, and the participant restarted with the shortest path and slowest competitor	• Trial duration ICF-CY domains: activities • Trial path length ICF-CY domains: not categorized	Both groups improved significantly Between groups comparison: significant differences for the DCD group compared to the TD group were seen
Snapp-childs et al. [60] Cross-over experimental study	51 children with DCD aged 5–11 years They were divided into two groups and two subgroups (according to age): Group 1: A. Younger group (n = 11 boys) B. Younger (n = 10; 8 boys) Group 2: A. Older (n = 12; 7 boys) B. Older (n = 14; 6 boys)	≤ 16	Phantom Omni	3D tracing tasks: the task was to push a (virtual) brightly coloured fish along a curved path from a starting to a finishing point while racing a competitor fish. They were instructed to (1) trace the entire path with their fish, while (2) racing and ‘beating’ a competitor fish that took 20 s to travel the path from start to finish. Trials were automatically terminated after 90 s if the child failed to complete the path 2D drawing tasks: the task was to view a figure, then to copy (not trace) the figure on the computer screen using a handheld stylus in the dominant hand. Participants completed two repetitions of each of nine forms for a total of eighteen trials	• Trial duration ICF-CY domains: activities	3D tracing task: the younger children exhibited less improvement then did the older children. However, no significant difference was found 2D drawing tasks: older children produced figures with less error compared to younger children. However, no significant difference was found Comparing between tasks: no significant difference was found
Soares et al. [53] Case study	1 boy with DCD aged 8 years old	≤ 15	Microsoft Kinect	Each week used different games with a total of 15 games	• MABC-2 ICF-CY domains: activities • DCD-Q ICF-CY domains: activities • COPM ICF-CY domains: activities and participation	• All outcome measurements were increased significantly
Straker et al. [57] Cross-over experimental study	21 children with DCD (10 boys), aged 9–12 years old They were randomly assigned to either: A. VR group (n = 10) B. Usual care (n = 11)	≤ 16	Sony PlayStation 3 and Microsoft Kinect	Group A: children were encouraged to play a variety of the games on both consoles Group B: children continued using traditional/sedentary games throughout the study	• MABC-2 ICF-CY domains: activities • Kinematic performance ICF-CY domains: not categorized • DCD-Q ICF-CY domains: activities	• No significant difference between groups for any of the outcome measurements
Wattad et al. [58] Quasi experimental study	30 children aged 4–6 years They were assigned to either: A. DCD group (n = 10; 4 boys) B. TD group (n = 20; 10 boys)	≤ 16	Timocco	Each child played the “Falling Fruit” game. During this game, the virtual monkey has to catch a piece of fruit dropping from the top of the screen, and place it in one of two baskets, set near the legs of the monkey	• MABC-2 ICF-CY domains: activities • VR game score ICF-CY domains activities • Hand path length ICF-CY domains: not categorized	There were statistically significant differences in the MABC-2 and the game score between the two groups in favour of the control group. However, there were no statistically significant differences in the path length of each hand between the two groups

*ICF-CY* international classification of functioning, disability and health children and youth, *RCT* randomized controlled trial, *DCD* developmental coordination disorder, *VR* virtual reality, *MABC-2* movement assessment battery for children-2, *TD* typical development, *TFT* task- oriented functional training; *DCD-Q* developmental coordination disorder questionnaire, *COPM* Canadian occupational performance measure, *FSM* functional strength measure, *HHD* hand-held dynamometer; *TST* task-specific training, *NTT* neuromotor task training, *PERF-FIT* performance and fitness battery, *BOT-2* Bruininks-Oseretsky test of motor proficiency, second edition

## Results

### Study selection

**Fig. 1:**
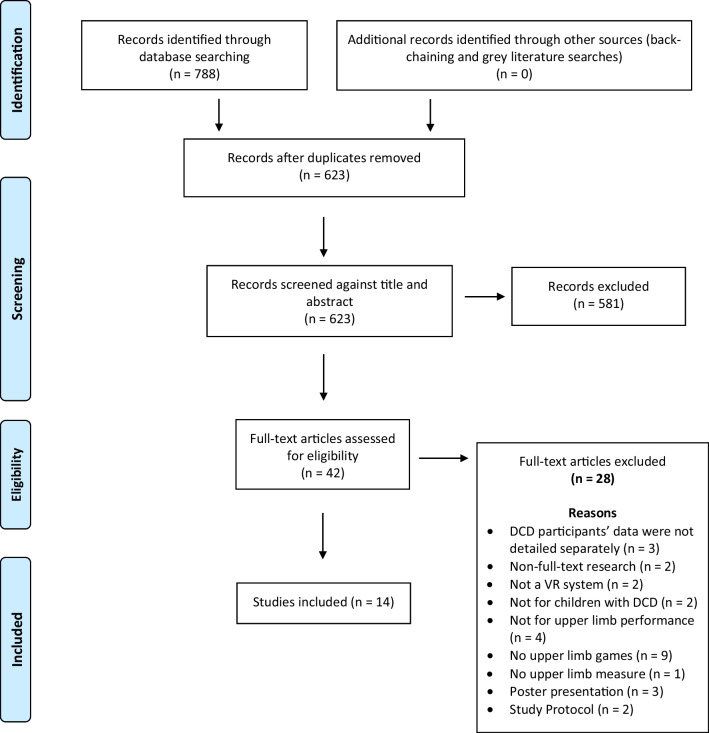
RISMA flow diagram of included studies

Seven hundred and eighty-eight studies were identified through the systematic search of the databases. Six hundred and twenty-three articles were screened for their titles and abstracts after eliminating duplicates, out of which 42 studies were evaluated in full text. Out of these, 14 studies that fulfilled the inclusion criteria were included in the review. The selection process is illustrated by the PRISMA flow diagram in Fig. 1.

### Study characteristics

Table 1 provides an overview of the key features of the 14 studies included in the current scoping review, which collectively examined 356 children and adolescents with DCD aged 4 to 16 years (mean age = 10 years). Just under the half of the participants were boys (n = 172), although one study [47] was excluded from this count as it combined data for typically developing boys with those diagnosed with DCD. Publication years ranged from 2013 to 2023 and all were published within the last 10 years.

#### Study designs

In terms of study design, Randomized Controlled Trials (RCTs) were the most common, with six studies using this design to examine a total of 204 children and adolescents with DCD. Additionally, there were four Quasi-Experimental studies, two Cross-Over Experimental designs, one Pilot Feasibility study, and one Case study.

#### Tools used to measure the upper limb motor performance

In our review, we aimed to categorize the outcome measures used to assess the impact of VR interventions on upper limb motor performance in children and adolescents with DCD, as detailed in Table 2, into the established ICF-CY domains: (1) body functions and structures, (2) activities and (3) participation. This classification was intended to align the measures with a globally recognized framework for health and disability. However, during the initial literature review, we noted that there was insufficient evidence from, and precedent in, the existing literature to support a straightforward classification of outcome measures within these ICF-CY domains alone. Consequently, we created an additional domain which combined activities and participation into a coherent multidomain, based on the American Physical Therapy Association Pediatrics (APTA) guidelines [48] and other sources [49]. Thus, the outcome measures were organized into four principal domains: (1) body functions and structures, (2) activities, (3) participation, and (4) a multidomain covering both activities and participation.

**Table 2:**
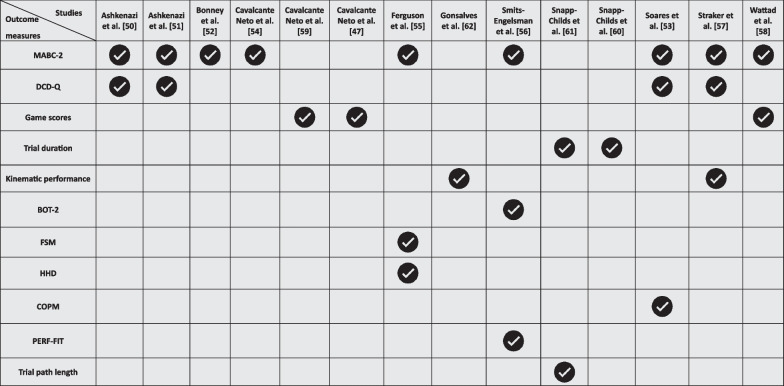
Exploring the Selection of Outcome Measures in Included Studies

*MABC-2* movement assessment battery for children-2, *DCD-Q* developmental coordination disorder questionnaire, *BOT-2* Bruininks-Oseretsky test of motor proficiency, second edition, *FSM* functional strength measure; *HHD* hand-held dynamometer; *COPM* Canadian occupational performance measure, *PERF-FIT* performance and fitness battery

#### Body functions and structures

No study included outcome measures specifically targeting the body functions and structures domain of the ICF-CY.

#### Activities

Outcome measures related to the ‘Activities’ domain of the ICF-CY were predominantly used in the studies reviewed, with the MABC-2 being the primary outcome measure in approximately two-thirds of the studies [50–58]. This underscores the significant role of this tests in evaluating the motor skills of children and young people with DCD. The DCD-Q featured in four studies [50, 51, 53, 57]. In addition, various other instruments were employed, including game scores [47, 59], trial duration [60, 61], and kinematic performance [57, 62]. Furthermore, studies also utilized the Functional Strength Measure (FSM) [55], Hand-Held Dynamometry (HHD) [55], the BOT-2 [56], Performance and Fitness (PERF-FIT) battery scores [56], and trial path length [61].

#### Participation

Only one study [52] included outcome measures related to participation, utilizing the Children's Self‐perceptions of Adequacy in and Predilection for Physical Activity (CSAPPA) questionnaire and the Participation in Activities of Daily Living for Adolescents’ Questionnaire (PADLA-Q). As this survey does not relate to upper-limb performance, it was not included in Table 1.

#### Multidomain (activities and participation)

Only one study [53] included outcome measures related to activities and participation, specifically employing the Canadian Occupational Performance Measure (COPM).

### The VR system/equipment utilized

With respect to the VR instruments employed, it was observed that all the included studies employed nVR, with no studies using iVR (see Table 3). Notably, the majority of these studies [47, 50–57, 59, 62] utilized non-specific (i.e., commercial) nVR platforms, such as Nintendo Wii, Sony PlayStation 2 and 3, EyeToy, and Microsoft Kinect. Specific nVR systems designed for rehabilitation (e.g., Timocco) were implemented in three studies [58, 60, 61].

**Table 3:**
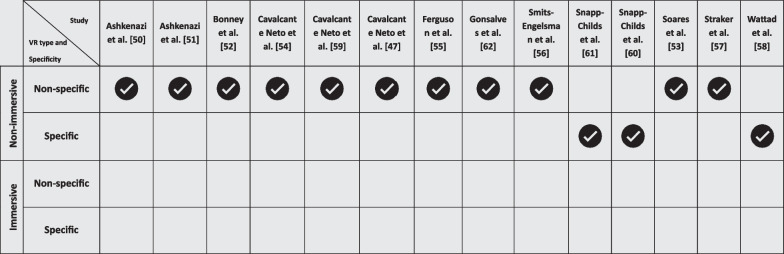
Overview of VR Equipment Utilized in Included Studies

### Distribution of studies by upper limb focus

All included studies assessed the impact of VR on upper extremity function. However, it is noteworthy that only four of these studies [58, 60–62] were specifically designed to target upper limb motor performance.

### Impacts of VR on upper limb motor performance

Most of the included studies [47, 50–52, 54–56, 58, 60, 62] (refer to Table 4) reported no significant improvement in upper limb motor performance for children and adolescents with DCD when using VR interventions. However, two studies [53, 61] documented significant improvements in all measured outcomes, and two other studies [57, 59] observed significant improvements in specific tasks.

**Table 4:**
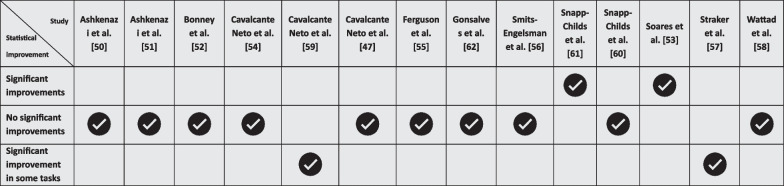
Impacts of VR on upper limb motor performance

Of the four studies that directly targeted upper limb motor performance, three [58, 60, 61] found no improvement using specific nVR systems. In contrast, the only study [62] that reported improvements employed a non-specific nVR system, using Sony PlayStation 3 Move and Microsoft Kinect.

### Enjoyment and motivation

In addition to any improvements in measures of performance, it is important to evaluate the enjoyability of an intervention, particularly in the context of interventions aimed at children [63]. In four studies [47, 50, 52, 56], enjoyment and motivation were directly assessed using three different tools: the Short Feedback Questionnaire for children (SFQ-Child) [50], the CSAPPA questionnaire [52] and the Enjoyment scale [47, 56]. In three of these studies [47, 50, 56], children and adolescents were reported to experience high enjoyment while engaging with VR technology in game-based activities. Furthermore, while not directly measuring enjoyment, two other studies [51, 61] noted that children and adolescents reported enjoying participating in the VR interventions. These findings suggest that VR interventions generally tend to be enjoyable for children and adolescents with DCD, which may contribute to improved motivation and engagement in therapeutic activities.

## Discussion

This scoping review aimed to explore the existing literature on VR interventions for upper limb motor performance in children and adolescents with DCD, focusing on identifying prevalent VR systems and sub-systems employed in these studies. Fourteen studies were reviewed, examining a total of 431 children and adolescents with DCD. Unlike other reviews which have drawn conclusions about general motor performance based on studies that focused predominantly on balance, rather than comprehensive motor skills, this scoping review focused on the impact of the VR interventions for upper limb motor performance in children and adolescents with DCD. Furthermore, this review is the first in this area to employ a technology taxonomy to distinguish between iVR and nVR systems. This more detailed assessment of technical characteristics may be critical to optimising the use of immersive technologies for motor development interventions.

### Study characteristics

Although the age range of participating children and adolescents spanned from 4 to 16 years old, 8 out of the 14 included studies focused on the age group of 7–10 years old, presumably because the size and ergonomics of VR technologies make them unsuitable for the smaller frames of younger children. Furthermore, the lack of focus on older children may limit generalizability for several reasons. Firstly, older children with DCD may have developed compensatory strategies or improved their motor skills over time, which may result in different intervention requirements compared to younger children [64, 65]. It is crucial to study older children separately to better understand their unique needs and how VR interventions can be tailored to address them effectively. Secondly, older children with DCD may have greater self-awareness of their motor challenges, which could influence their responsiveness to interventions [66]. Therefore, the variation in motor performance between older and younger children with DCD, as a result of their age-related differences in coping strategies and self-awareness, emphasizes the need for targeted research. Generalizing findings from one age group to another may not accurately reflect the efficacy of VR interventions in improving upper limb motor performance for all children with DCD.

### Study designs

In our review, six RCTs were included. RCTs are widely recognized as offering the most potentially robust form of evidence in clinical research [67], which is crucial for developing practice guidelines, clinical recommendations, and for informing practical applications in various areas of healthcare [68]. However, it is noteworthy that the majority of the studies (n = 8) in our review were categorized as levels III and IV in terms of evidence hierarchy, indicating they are susceptible to threats to internal validity and may exhibit a higher degree of bias in their results compared to RCTs [69].

Given this distribution of evidence levels, the impact and generalizability of the interventions examined in our scoping review must be considered with caution. While the findings from these studies are promising and suggest potential benefits for future applications, their generalization is limited without further validation through more rigorous research designs. Therefore, future studies with a stronger methodological approach, particularly those employing higher-tier evidence designs such as RCTs, are essential to affirm the efficacy and applicability of VR interventions in improving upper limb motor performance in children and adolescents with DCD.

### Tools used to measure upper limb motor performance

This review categorized the outcome measures used in the studies according to the ICF-CY domains, focusing specifically on body functions and structures, activities, and participation. This alignment with the ICF-CY framework allows for a more structured analysis of how VR interventions impact upper limb motor performance in children and adolescents with DCD.

#### Body functions and structures

Based on the resources we used for categorization, it was found that the included studies did not explicitly measure changes in 'Body Functions and Structures'. The assessment of 'Body Functions' is crucial, as it pertains directly to the physiological functions of body systems involved in motor planning and execution—areas where children and adolescents with DCD typically experience significant difficulties [70, 71].

In children and adolescents with DCD, the primary challenges are rooted in motor planning and execution [70]. Motor planning or praxis is the ability to organize, plan, and execute motor tasks. This involves not just the conceptualization of the task but also the ability to physically carry out the associated movements in a coordinated way [71]. For children and adolescents with DCD, there can be a significant disconnect between knowing how to perform a task and executing it effectively [9]. This discrepancy can affect a wide range of activities—from simple tasks like buttoning a shirt to more complex kinematic sequences like playing sports.

Moreover, these difficulties in motor planning and execution are often observed as poor coordination, delays in reaching motor milestones, and clumsiness, all of which are presumed to be symptomatic of underlying dysfunctions in motor programming and neuromotor execution [9]. These aspects of motor dysfunction in DCD can significantly impact daily activities, reducing the impact with which these children and adolescents engage in both basic and complex tasks.

In examining the landscape of research on interventions for children and adolescents with DCD, our scoping review has identified differences in how outcome measures are categorized, particularly in the 'Body Functions and Structures' domain. Previous reviews [34, 72] have included studies that categorize outcome measures such as the MABC-2 and the BOT-2 under this domain, which contrasts with the official classifications by the American Physical Therapy Association Pediatrics Section [48]. These classifications typically reserve the 'Body Functions and Structures' category for direct measures of physiological functions, such as aerobic fitness evaluated through the 6-min walk test, or anaerobic performance as assessed by the Muscle Power Sprint Test. Our review, however, found no studies that explicitly categorized their outcomes under 'Body Functions and Structures' for measures traditionally associated with upper limb function. Some studies included measures that could potentially fit under anaerobic performance, such as HHD [55] and kinematic performance assessments [57, 62]. Nonetheless, we chose not to categorize these measures under 'Body Functions and Structures' due to the lack of consensus in the existing APTA Pediatrics regarding their classification [48]. This decision reflects a cautious approach to categorization, aiming to maintain consistency and clarity in how outcomes are reported and interpreted within the context of VR interventions for DCD.

#### Activities

The majority of outcome measures were concentrated on the 'Activities' domain, reflecting direct engagement with tasks and actions. Similar to our findings, Mentiplay et al. [34] also reported a majority of studies using outcome measures in the activities domain of the ICF-CY. The MABC-2, used in about two-thirds of the studies, plays a crucial role in evaluating motor skills by measuring the performance of specific tasks that are indicative of upper limb function. This outcome is unsurprising, as this battery is a reliable and valid measure to assess motor competence in children and adolescents with DCD [10], and as such is prevalent in this research field. Several studies also used Nintendo Wii game scores [47, 59] or trial duration [60, 61] as a primary measure to assess movement performance in children and adolescents with DCD, which warrants careful consideration. Although the interactive nature of nVR games can be engaging and might seem to offer a direct assessment of motor skills, there are significant concerns regarding the accuracy and comprehensiveness of this approach. The scoring system and the duration of game trials in these games is primarily designed for entertainment rather than clinical assessment [73], and these metrics are unlikely to fully capture the complexity and range of motor difficulties experienced by children and adolescents with DCD. Game scores might reflect only a limited aspect of motor ability, focusing on specific movements and outcomes, whereas trial duration could vary significantly due to factors such as game familiarity, individual learning curves, and the child's ability to adapt to the game mechanics [74]. Moreover, children and adolescents with DCD might employ compensatory strategies to complete tasks, which can affect both their scores and the time taken to complete trials, leading to potentially misleading interpretations of their motor skills [75]. Therefore, while the use of Nintendo Wii games can contribute valuable insights, relying exclusively on game scores and trial durations for assessment can result in an incomplete understanding of the child's motor abilities. The development and validation of a comprehensive assessment tool that captures the nuances of upper limb motor performance in this population could help achieve a better understanding of the impact of VR interventions and facilitate comparisons across studies.

#### Multidomain and participation

Our review found that only a single study [53] explored measures spanning both 'Activities' and 'Participation' domains, employing the COPM. This tool assesses the child's perceived performance of everyday activities and highlights the interconnectedness of activity competence and participation in daily life. This approach underscores the recognition of how motor skill enhancements can have broader impacts, not just on task execution but also on overall life involvement.

However, it was notable that only one other study [52] focused explicitly on the 'Participation' domain itself, utilizing tools such as the CSAPPA questionnaire and the PADLA-Q. These measures are pivotal as they provide direct insights into how improved motor functions translate into real-world outcomes. Such assessments can reveal whether enhancements in motor skills lead to greater involvement in school activities or social interactions, which are critical aspects of a child's development and quality of life. Interestingly, our findings align with those of a previous review, which identified only two studies assessing the participation domain, one of which Bonny et al. [52] is included in our review. The other study, by Howie et al. [76], further underscores the limited but growing attention to this crucial aspect of DCD intervention research.

The limited focus on 'Participation' within the existing literature highlights a significant gap. Detailed evaluations in this domain are essential for understanding the full social implications of motor impairments and the true impact of interventions aimed at alleviating these challenges. Future research should prioritize the inclusion of participation-focused outcome measures to comprehensively assess how interventions influence the daily lives and social integration of children and adolescents with DCD.

### The VR system/equipment utilized

VR is understood as an umbrella term encompassing a diverse range of technologies [39]. VR systems have been classified based on their level of immersion into iVR and nVR [77]. Additionally, these systems are further categorized as 'specific' or 'non-specific' based on their intended use and design [39]. 'Specific' systems refer to those developed exclusively for rehabilitation purposes, tailored to meet therapeutic objectives [39]. In contrast, 'non-specific' systems encompass recreational and/or ‘off-the-shelf’ video games that were not originally designed with therapeutic goals in mind but have been adapted for use in such interventions [39]. This classification is crucial to understand the varied nature and potential of VR interventions in the context of improving upper limb motor performance in children and adolescents with DCD [39, 77].

#### Non-specific nVR systems

In a majority of the included studies (n = 11), researchers evaluated the impact of VR on upper limb motor performance in children and adolescents with DCD using non-specific nVR systems. These systems, which typically include commercial platforms such as the Nintendo Wii, Sony PlayStation, and Microsoft Kinect, were not originally designed with therapeutic goals for children and adolescents with DCD in mind [77]. Consequently, they may lack specific adjustments or features necessary to meet the nuanced therapeutic demands and objectives for this population [39, 77].

However, the attributes of nVR, such as low cost and portability, contribute to its growing popularity and accessibility in therapeutic settings [78]. The general accessibility and user-friendly nature of these systems make them an attractive option for clinical and home use [30]. Despite these benefits, the efficacy of non-specific nVR systems in addressing the specific challenges faced by children and adolescents with DCD remains an area requiring further investigation. The use of commercial games and activities, while engaging, might not adequately target the specific motor skills deficits characteristic of DCD, potentially limiting the therapeutic impact of such interventions.

#### Specific nVR systems

In contrast, a smaller subset of the studies [58, 60, 61] employed specific nVR systems designed or adapted for therapeutic purposes. These systems, which include bespoke VR technologies such as the Phantom Omni and Timocco, offer a more focused approach to addressing upper limb motor performance. By being specifically designed or adapted for therapeutic use, these systems potentially provide activities and tasks that are more directly aligned with the challenges faced by children and adolescents with DCD. As such, these specific nVR systems may offer more targeted and clinically relevant exercises and activities, incorporating motor skills training and feedback mechanisms that are tailored to the unique needs of this group. Such specificity in design and application might lead to more effective outcomes, particularly in the context of upper limb motor performance.

However, the limited number of studies using specific nVR systems indicates a gap in research and highlights the need for more extensive investigation into the efficacy of these tailored VR interventions. The potential of specific nVR systems to provide more focused and effective therapeutic experiences for children and adolescents with DCD warrants further exploration, particularly in comparison to the more commonly used non-specific nVR platforms.

#### iVR systems

One striking feature of this review is the total absence of iVR (specific or non-specific) interventions for upper limb function in children and adolescents with DCD. This lack of research, despite the recent opportunities with this technology, highlight an unexplored area in VR-based interventions for children and adolescents with DCD that may offer different, potentially more engaging experiences. In our review, we identified three studies, by the same author, utilizing specific iVR interventions [79–81]. However, these studies were not included in the final analysis because they did not provide detailed, separate data for participants with DCD. Instead, the data for these participants were combined with data from children and adolescents with CP. This aggregation of data across different conditions obscures specific outcomes and intervention efficacies for children and adolescents with DCD, thereby limiting the applicability of the findings to this particular group. While iVR has shown promise in other paediatric populations, such as those with upper limb motor impairment [82], the direct applicability of these findings to children and adolescents with DCD remains unclear.

The absence of iVR interventions for children and adolescents with DCD, as highlighted in this review, calls for more detailed and separate investigation in this area. Given the possible benefits of iVR in other paediatric conditions, future research could significantly contribute to the development of effective, engaging therapeutic options for children and adolescents with DCD, ultimately improving their functional outcomes and quality of life.

### Distribution of studies by upper limb focus

In our review, while all included studies assessed the impact of VR on upper extremity function, only four studies [58, 60–62] specifically targeted upper limb motor performance. This finding reflects a trend of DCD intervention research, where studies explicitly examining upper limb motor performance are relatively uncommon. Unlike research in populations such as children and adolescents with CP [30], neurological impairments [29], and Down syndrome [83], where the effects of VR on upper limb performance have been more extensively explored, studies concentrating on upper limb motor skills in children and adolescents with DCD are notably scarce. The majority of the studies included in our review primarily investigated VR’s impact on balance, with upper extremity coordination being considered a secondary outcome. This approach is exemplified by the frequent use of the Nintendo Wii balance board in VR interventions, such as in the study by Bonney et al. [52], which offered 26 Wii games but only four games that directly engaged upper limb motor skills. This distribution highlights a research emphasis on balance over specific upper limb skills in DCD intervention studies, contrasting with the more diverse focus seen in research involving other paediatric populations. Therefore, the interpretation of the efficacy of VR interventions for improving upper extremity outcomes in children and adolescents with DCD should be contextualized within this wider landscape. The limited focus on upper limb motor performance in existing DCD studies underscores an important area for future research, highlighting the need for more targeted investigations to address this gap, particularly in light of the more extensive research conducted in other paediatric populations.

### Impact of VR on upper limb motor performance

A critical aspect of our review focuses on the impact of VR interventions on upper limb motor performance in children and adolescents with DCD. Interestingly, most studies [47, 50–52, 54–56, 58, 60, 62] reported no significant improvement in upper limb motor performance following VR interventions. This finding suggests that while VR technology is increasingly being explored for therapeutic purposes, its impact on enhancing upper limb motor skills in children and adolescents with DCD is not consistently demonstrated.

However, there were notable exceptions. Two studies [53, 61] documented significant improvements across all measured outcomes. Additionally, two other studies [57, 59] observed significant improvements in specific tasks. The variability in outcomes indicates that the efficacy of VR interventions may depend on several factors, including the design of the VR intervention, the specific tasks and games involved, and individual differences among the children and adolescents with DCD.

Particularly revealing is the observation that among the four studies specifically targeting upper limb motor performance, three [58, 60, 61] found no improvement using specific nVR systems. In contrast, the only study [62] that reported improvements used a non-specific nVR system. This outcome raises questions about the types of VR systems and their content that might be most beneficial for improving motor skills in children and adolescents with DCD. It suggests that the impact of VR interventions might not solely hinge on the immersive qualities of the technology but also on how well the activities and games are tailored to the specific needs of this population.

### Enjoyment and motivation

Five studies [47, 50, 51, 56, 61] in the review found that children and adolescents generally experienced high levels of enjoyment while engaging with VR technology in game-based activities which is a crucial aspect to consider. Enjoyment is known to be an essential factor in promoting adherence and engagement in therapeutic interventions [84]. This enjoyment might suggest that these interventions are more likely to be practiced outside of the clinical setting, such as at home. The use of VR interventions that are enjoyable and motivating for children and adolescents with DCD could potentially lead to improved motor performance outcomes in the long term. Nevertheless, more research is needed to explore the relationship between enjoyment, motivation, and therapeutic outcomes in this context.

### Implications for future research and clinical practice

Our scoping review has identified a clear need for further rigorous research into the use of VR interventions for impacting upper limb motor performance in children and adolescents with DCD. Despite the potential shown by VR technologies, the body of research remains relatively small and methodologically diverse. There is a crucial requirement for more comprehensive studies that employ standardized assessment tools and larger sample sizes to assess upper motor performance for children and adolescents with DCD. These studies should aim to validate and extend the findings reported, ensuring a stronger evidence base from which clinical practices can be developed.

A notable gap in the literature is the emphasis on the 'Activities' domain of the ICF-CY, with a lack of focus on the body functions and structures and participation domains. Future research should consider including outcome measures that assess the translation of motor skills improvements into enhanced participation in daily life activities. This approach is vital for evaluating the real-world applicability of VR interventions and for validating their impact on the quality of life of children and adolescents with DCD.

Lastly, there is a clear need for longitudinal studies that investigate the long-term effects of VR interventions. Understanding whether improvements gained through VR are sustained over time is essential for developing ongoing support strategies that can adapt to the evolving needs of children and adolescents with DCD.

By addressing these areas in future research, researchers can provide more definitive guidance to practitioners on the likely impact of VR interventions, ultimately leading to improved outcomes for children and adolescents with DCD in various aspects of their lives. This comprehensive approach ensures that VR can be an integral part of the therapeutic landscape for children and adolescents with developmental challenges.

## Limitations

Our scoping review faced certain limitations that may have influenced the breadth and depth of the conclusions drawn. Firstly, the review was restricted to studies published in English. This language limitation may have excluded relevant studies published in other languages, potentially introducing language bias and reducing the comprehensiveness and generalizability of our findings. Secondly, not all outcome measures identified in the reviewed studies were categorizable based on the ICF-CY domains as outlined in the American Physical Therapy Association (APTA) Pediatrics [48]. Consequently, our ability to discuss findings according to these domains was restricted, which might have impacted the depth of analysis concerning the specific impacts of VR interventions on various aspects of functioning and performance in children and adolescents with DCD.

## Conclusion

In this scoping review, which aimed to explore the use of VR for improving upper limb motor performance in children and adolescents with DCD, we found that the range and nature of VR interventions are diverse and centred around nVR systems. These interventions commonly employed commercial (non-specific) gaming platforms such as Nintendo Wii, Sony PlayStation, and Microsoft Kinect, indicating a focus on accessible technology. However, the review highlighted a notable gap in the use of iVR interventions, with a total absence of studies using fully immersive systems, suggesting unexplored potential in this area. The majority of VR interventions aimed at general motor coordination or balance, with only a small subset directly targeting upper limb motor performance. This finding aligns with a broader trend in DCD research, where specific focus on upper limb motor skills is relatively scarce.

The measurement tools used to assess these VR interventions varied, with motor competence measures such as the MABC-2 and DCD-Q being the most common. However, the efficacy of using game scores and trial durations as primary measures for assessing upper limb motor performance in children and adolescents with DCD was questioned, raising concerns about their accuracy and comprehensiveness. This underscores the need for more targeted and clinically relevant measurement tools in future research. A notable gap in the literature is the emphasis on the 'Activities' domain of the ICF-CY, with limited focus on the 'Body Functions and Structures' and 'Participation' domains. This imbalance suggests that future studies should consider a broader range of outcome measures to ensure that all aspects of upper limb motor performance are adequately addressed, from underlying physiological functions to real-world participation in daily life and social activities.

### Supplementary Information

Supplementary Material 1. Table S1. Terms used on database search.

Supplementary Material 2.Table S2. Sources excluded following full-text review

Supplementary Material 3.Table S3. Data extraction instrument

Supplementary Material 4.Table S4. List of Abbreviations

## Data Availability

All data generated or analysed during this study are included in this published article (and its supplementary files).
