# Supplementary material for: Evaluating the impact of virtual reality game training on upper limb motor performance in children and adolescents with developmental coordination disorder: a scoping review using the ICF framework
Source: J Neuroeng Rehabil. 2024 Jun 5;21:95. doi: 10.1186/s12984-024-01393-y (PMC11151681; doi:10.1186/s12984-024-01393-y)
Supplement: Supplementary file 1 — Supplementary Material 1. Table S1. Terms used on database search. [file 12984_2024_1393_MOESM1_ESM.docx]

**Additional file 1. Table S1. Terms used on database search.**

| Database | Search format | Items found |
| --- | --- | --- |
| PUBMED | #1  Search ((((((((((((“Virtual reality”) OR VR) OR immersive) OR IVR) OR "head-mounted display") OR headset) OR Nintendo) OR Wii) OR Nintendo Wii-Fit) OR Kinect) OR PlayStation) OR X-BOX) OR exergame) OR Non-immersive (171425)  #2  Search (((((((child) OR p?ediatr*) OR boy) OR girl) OR young) OR "young people") OR "young person") OR adolescent (3982078)  #3  Search ("Developmental coordination disorder" OR DCD OR dyspraxia (13367)  #4  Search (((((((Therapy) OR rehabilitation) OR training) OR performance) OR motor) OR "functional training") OR exercise) OR intervention (8375781)  #5  Search ((((((((((((((((Virtual reality) OR VR) OR immersive) OR IVR) OR "head-mounted display") OR headset) OR Nintendo) OR Wii) OR Nintendo Wii-Fit) OR Kinect) OR PlayStation) OR X-BOX) OR exergame) OR Non-immersive)) AND ((((((((child) OR p?ediatr*) OR boy) OR girl) OR young) OR "young people") OR "young person") OR adolescent)) AND ((("Developmental coordination disorder") OR DCD) OR dyspraxia)) AND ((((((((Therapy) OR rehabilitation) OR training) OR performance) OR motor) OR "functional training") OR exercise) OR intervention) (529) | 529 |
| EMBASE  via Ovid | 1. ("Virtual reality" or VR or immersive or IVR or "head-mounted display" or headset or Nintendo or "Nintendo Wii-Fit" or Wii or PlayStation or Kinect or X-BOX or exergame or Non-immersive). (93686)  2. (Child or children or p?ediatr* or boy or girl or young or "young person" or "young people" or adolescent). (6293589)  3. ("Developmental coordination disorder" or DCD or dyspraxia). (10914)  4. (Therapy or rehabilitation or training or performance or motor or "functional training" or exercise or intervention). (13833424)  5. 1 and 2 and 3 and 4 (62) | 62 |
| MEDLINE  via Ovid | 1. ("Virtual reality" or VR or immersive or IVR or "head-mounted display" or headset or Nintendo or "Nintendo Wii-Fit" or Wii or PlayStation or Kinect or X-BOX or exergame or Non-immersive). (39309)  2. (Child or p?ediatr* or boy or girl or young or "young person" or "young people" or adolescent). (5492033)  3. ("Developmental coordination disorder" or DCD or dyspraxia). (5019)  4. (Therapy or rehabilitation or training or performance or motor or "functional training" or exercise or intervention). (7814788)  5. 1 and 2 and 3 and 4 (37) | 37 |
| CINAHL Ultimate  Via EBSCOhost | #S1. "Virtual reality" OR VR OR IVR OR immersive OR "head-mounted display" OR headset OR Nintendo OR "Nintendo Wii-Fit" OR Wii OR PlayStation OR Kinect OR exergame OR Non-immersive (17,426)  #S2. Child OR Children OR p?ediatr* OR boy OR girl OR young OR "young person" OR "young people" OR adolescent (1,340,331)  #S3. "Developmental coordination disorder" OR DCD OR dyspraxia (4044)  #S4. Therapy OR rehabilitation OR training OR performance OR motor OR "functional training" OR exercise OR intervention (2,744,288)  #S5. (therapy OR rehabilitation OR training OR performance OR (functional training or functional exercises) OR exercise OR intervention*) AND (S1 AND S2 AND S3 AND S4) (42) | 42 |
| Web of Science | 1. "Virtual reality" (All Fields) or VR (All Fields) or IVR (All Fields) or immersive (All Fields) or "head-mounted display" (All Fields) or headset (All Fields) or Nintendo (All Fields) or "Nintendo Wii-Fit" (All Fields) or Wii (All Fields) or PlayStation (All Fields) or Kinect (All Fields) or exergame (All Fields) or Non-immersive (All Fields) or X-BOX (All Fields) (232645)  2. Child (All Fields) or children (All Fields) or p?ediatr* (All Fields) or boy (All Fields) or girl (All Fields) or young (All Fields) or "young person" (All Fields) or "young people" (All Fields) or adolescent (All Fields) (5026776)  3. "Developmental coordination disorder" (All Fields) or DCD (All Fields) or dyspraxia (All Fields) (10080)  4. Therapy (All Fields) or rehabilitation (All Fields) or training (All Fields) or performance (All Fields) or motor (All Fields) or "functional training" (All Fields) or exercise (All Fields) or intervention (All Fields) (13458430)  5. #1 AND #2 AND #3 AND #4 (89) | 89 |
| Cochrane | All Text  ('Developmental coordination disorder') OR (DCD) OR (dyspraxia)  AND  Title Abstract Keyword  (Virtual reality) OR (VR) OR (immersive) OR (IVR) OR ('head-mounted display') OR (headset) OR (augmented reality) OR (exergame) OR ('Nintendo Wii-Fit') OR (Wii) OR (Kinect)  AND  Title Abstract Keyword  (Child) OR (Children) OR (p?ediatr*) OR (boy*s) OR (girl*s) OR (young) OR (young people) OR (adolescent)  AND  Title Abstract Keyword  (Therapy) OR (rehabilitation) OR (training) OR (performance) OR ('functional training') OR ('exercise') OR (intervention) | 29 |
| Google Scholar | Virtual Reality and children with Developmental Coordination Disorder OR Dyspraxia | 0 |
