# Supplementary material for: Evaluating the impact of virtual reality game training on upper limb motor performance in children and adolescents with developmental coordination disorder: a scoping review using the ICF framework
Source: J Neuroeng Rehabil. 2024 Jun 5;21:95. doi: 10.1186/s12984-024-01393-y (PMC11151681; doi:10.1186/s12984-024-01393-y)
Supplement: Supplementary file 2 — Supplementary Material 2.Table S2. Sources excluded following full-text review [file 12984_2024_1393_MOESM2_ESM.docx]

**Additional file 2. Table S2. Sources excluded following full-text review**

| **N** | **Author** | **Reason** |
| --- | --- | --- |
| **1** | Ashkenazi, et al. 2011 ^1^ | Poster presentation |
| **2** | Avila-Pesantez, et al. 2018 ^2^ | Not a VR system |
| **3** | Bonney, et al. 2017 ^3^ | No upper limb games |
| **4** | Bonney, et al. 2017 ^4^ | No upper limb games |
| **5** | Bortone, et al. 2017 ^5^ | DCD participants data was not detailed separately |
| **6** | Bortone, et al. 2018 ^6^ | DCD participants data was not detailed separately |
| **7** | Bortone, et al. 2020 ^7^ | DCD participants data was not detailed separately |
| **8** | Cavalcante Neto, et al. 2019 ^8^ | Study Protocol |
| **9** | EbrahimiSani, et al. 2020 ^9^ | Not for upper limb performance |
| **10** | Engel-Yeger, et al. 2017 ^10^ | No upper limb games |
| **11** | Ganapathy Sankar, et al. 2020 ^11^ | Non-full-text research |
| **12** | Hammond, et al. 2013 ^12^ | No upper limb games |
| **13** | Howie, et al. 2016 ^13^ | Not for upper limb performance |
| **14** | Howie, et al. 2017 ^14^ | Not for upper limb performance |
| **15** | Jelsma, et al. 2019 ^15^ | No upper limb games |
| **16** | Jelsma, et al. 2021 ^16^ | No upper limb games |
| **17** | Jelsma, et al. 2023 ^17^ | No upper limb measure |
| **18** | Kang, et al. 2022 ^18^ | Not a VR system |
| **19** | Park, et al. 2022 ^19^ | Not for upper limb performance |
| **20** | Phelan, et al. 2023 ^20^ | Not for children with DCD |
| **21** | Silva, et al. 2021 ^21^ | Not for children with DCD |
| **22** | Smits-Engelsman, et al. 2015 ^22^ | No upper limb games |
| **23** | Smits-Engelsman, et al. 2017 ^23^ | No upper limb games |
| **24** | Smits-Engelsman, et al. 2020 ^24^ | No upper limb games |
| **25** | Straker, et al. 2011 ^25^ | Study Protocol |
| **26** | Straker, et al. 2015 ^26^ | Poster presentation |
| **27** | Tarakci, et al. 2018 ^27^ | Poster presentation |
| **28** | Tresser 2012 ^28^ | Non-full-text research |

**Reference:**

1. Ashkenazi T, et al. Low cost virtual reality intervention program for children with developmental coordination disorder; three case studies. *Physiotherapy*. 2011;97

2. Avila-Pesantez D, et al. Athynos: Helping children with dyspraxia through an augmented reality serious game. IEEE; 2018:286-290.

3. Bonney E, et al. Learning better by repetition or variation? Is transfer at odds with task specific training? *PLoS One*. 2017;12(3):e0174214.

4. Bonney E, et al. Variable training does not lead to better motor learning compared to repetitive training in children with and without DCD when exposed to active video games. *Research in developmental disabilities*. 2017;62:124-136.

5. Bortone I, et al. Integration of serious games and wearable haptic interfaces for Neuro Rehabilitation of children with movement disorders: a feasibility study. IEEE; 2017:1094-1099.

6. Bortone I, et al. Wearable haptics and immersive virtual reality rehabilitation training in children with neuromotor impairments. *IEEE Transactions on Neural Systems and Rehabilitation Engineering*. 2018;26(7):1469-1478.

7. Bortone I, et al. Immersive virtual environments and wearable haptic devices in rehabilitation of children with neuromotor impairments: a single-blind randomized controlled crossover pilot study. *Journal of neuroengineering and rehabilitation*. 2020;17(1):1-14.

8. Cavalcante Neto JL, et al. Motor intervention with and without Nintendo® Wii for children with developmental coordination disorder: protocol for a randomized clinical trial. *Trials*. 2019;20(1):1-12.

9. EbrahimiSani S, et al. Effects of virtual reality training intervention on predictive motor control of children with DCD–A randomized controlled trial. *Research in developmental disabilities*. 2020;107:103768.

10. Engel-Yeger B, et al. Relationship between perceived competence and performance during real and virtual motor tasks by children with developmental coordination disorder. *Disability and Rehabilitation: Assistive Technology*. 2017;12(7):752-757.

11. Ganapathy Sankar U, Monisha R. Effectiveness Of Two Task-Oriented Interventions Over Cardiorespiratory Fitness And Motor Performance In Children With Developmental Coordination Disorder (DCD)-A Pilot Study. *International Journal of Research in Pharmaceutical Sciences*. 2020;11(4):6399-6403.

12. Hammond J, et al. An investigation of the impact of regular use of the W ii F it to improve motor and psychosocial outcomes in children with movement difficulties: a pilot study. *Child: care, health and development*. 2013;40(2):165-175.

13. Howie EK, et al. An active video game intervention does not improve physical activity and sedentary time of children at‐risk for developmental coordination disorder: a crossover randomized trial. *Child: care, health and development*. 2016;42(2):253-260.

14. Howie EK, et al. Understanding why an active video game intervention did not improve motor skill and physical activity in children with developmental coordination disorder: A quantity or quality issue? *Research in Developmental Disabilities*. 2017;60:1-12.

15. Jelsma L, et al. Movement control strategies in a dynamic balance task in children with and without developmental coordination disorder. *Journal of motor behavior*. 2019;

16. Jelsma L, et al. Effect of dual tasking on a dynamic balance task in children with and without DCD. *Human movement science*. 2021;79:102859.

17. Jelsma LD, et al. Type of active video-games training does not impact the effect on balance and agility in children with and without developmental coordination disorder: A randomized comparator-controlled trial. *Applied Neuropsychology: Child*. 2023;12(1):64-73.

18. Kang H-Y, et al. Effects of Augmented Reality-Based Dual-Task Program on Physical Ability by Cognitive Stage with Developmental Disabilities. MDPI; 2022:2067.

19. Park S-B, et al. Effect of a Cognitive Function and Social Skills-Based Digital Exercise Therapy Using IoT on Motor Coordination in Children with Intellectual and Developmental Disability. *International Journal of Environmental Research and Public Health*. 2022;19(24):16499.

20. Phelan I, et al. Home-based immersive virtual reality physical rehabilitation in paediatric patients for upper limb motor impairment: a feasibility study. *Virtual Reality*. 2023:1-16.

21. Silva TDd, et al. Comparison between conventional intervention and Non-immersive virtual reality in the rehabilitation of individuals in an inpatient unit for the treatment of COVID-19: a study protocol for a randomized controlled crossover trial. *Frontiers in psychology*. 2021;12:622618.

22. Smits-Engelsman BC, et al. Motor learning: an analysis of 100 trials of a ski slalom game in children with and without developmental coordination disorder. *PloS one*. 2015;10(10):e0140470.

23. Smits-Engelsman BCM, et al. The effect of exergames on functional strength, anaerobic fitness, balance and agility in children with and without motor coordination difficulties living in low-income communities. *HUMAN MOVEMENT SCIENCE*. OCT 2017;55:327-337. doi:10.1016/j.humov.2016.07.006

24. Smits-Engelsman B, et al. Motor skill learning in children with and without Developmental Coordination Disorder. *Human Movement Science*. 2020;74:102687.

25. Straker LM, et al. Rationale, design and methods for a randomised and controlled trial of the impact of virtual reality games on motor competence, physical activity, and mental health in children with developmental coordination disorder. *BMC Public Health*. 2011/08/18 2011;11(1):654. doi:10.1186/1471-2458-11-654

26. Straker L, et al. Can active video games enhance motor coordination in children with developmental coordination disorder? *Physiotherapy*. 2015;101:e680.

27. Tarakci E, et al. The effectiveness of Technotherapy applied with Xbox 360 KinectTM games in children with developmental coordination disability: a preliminary clinical study. *Developmental Medicine & Child Neurology*. 2018;60(S2):28-74. doi:<https://doi.org/10.1111/dmcn.13790>

28. Tresser S. Case study: Using a novel virtual reality computer game for occupational therapy intervention. *Presence*. 2012;21(3):359-371.
