# Supplementary material for: Evaluating the impact of virtual reality game training on upper limb motor performance in children and adolescents with developmental coordination disorder: a scoping review using the ICF framework
Source: J Neuroeng Rehabil. 2024 Jun 5;21:95. doi: 10.1186/s12984-024-01393-y (PMC11151681; doi:10.1186/s12984-024-01393-y)
Supplement: Supplementary file 3 — Supplementary Material 3.Table S3. Data extraction instrument [file 12984_2024_1393_MOESM3_ESM.docx]

**Additional file 3. Table S3. Data extraction instrument**

| **N** | **Author(s) and year of publication/Design** | **Study population, sample size and comparison groups** | **MABC-2 percentile** | **VR tool** | **Intervention protocols and comparator** | **Outcome measures**  **and ICF-CY domains** | **Results regarding upper limb motor performance** |
| --- | --- | --- | --- | --- | --- | --- | --- |
| 1 |  |  |  |  |  |  |  |
| 2 |  |  |  |  |  |  |  |
| 3 |  |  |  |  |  |  |  |
| .. |  |  |  |  |  |  |  |
