# Supplementary material for: Evaluating the impact of virtual reality game training on upper limb motor performance in children and adolescents with developmental coordination disorder: a scoping review using the ICF framework
Source: J Neuroeng Rehabil. 2024 Jun 5;21:95. doi: 10.1186/s12984-024-01393-y (PMC11151681; doi:10.1186/s12984-024-01393-y)
Supplement: Supplementary file 4 — Supplementary Material 4.Table S4. List of Abbreviations [file 12984_2024_1393_MOESM4_ESM.docx]

**Additional file 4. Table S4. List of Abbreviations**

ADLs Activities of Daily Living

BOT-2 Bruininks-Oseretsky test of motor proficiency, second edition

COPM Canadian Occupational Performance Measure

CP Cerebral Palsy

CSAPPA Children's Self-Perceptions of Adequacy in and Predilection for Physical Activity

DCD Developmental Coordination Disorder

DCD-Q Developmental Coordination Disorder Questionnaire

DSM-IV Diagnostic and Statistical Manual of Mental Disorders Fourth Edition

DSM-V Diagnostic and Statistical Manual of Mental Disorders Fifth Edition

FSM Functional Strength Measure

HHD Hand-Held Dynamometer

ICF-CY International Classification of Functioning, Disability and Health Children and

Youth

iVR immersive VR

MABC-2 Movement Assessment Battery for Children-2

NTT Neuromotor Task Training

nVR non-immersive VR

PADLA-Q Participation in Activities of Daily Living for Adolescents’ Questionnaire

PERF-FIT Performance and Fitness Battery

RCT Randomized Controlled Trial

SFQ-Child Short Feedback Questionnaire for children

TD Typical Development

TFT Task- Oriented Functional Training

TST Task-Specific Training

VR Virtual Reality
